# Voluntary control of auditory hallucinations: phenomenology to therapeutic implications

**DOI:** 10.1038/s41537-020-0106-8

**Published:** 2020-08-04

**Authors:** Ariel Swyer, Albert R. Powers

**Affiliations:** 1 Department of Behavioral Sciences, York College/CUNY, Jamaica, NY USA; 2 Department of Psychiatry and the Connecticut Mental Health Center, Yale University, New Haven, CT USA

**Keywords:** Psychosis, Psychosis, Biomarkers

## Abstract

Auditory verbal hallucinations (AVH) have traditionally been thought to be outside the influence of conscious control. However, recent work with voice hearers makes clear that both treatment-seeking and non-treatment-seeking voice hearers may exert varying degrees of control over their voices. Evidence suggests that this ability may be a key factor in determining health status, but little systematic examination of control in AVH has been carried out. This review provides an overview of the research examining control over AVH in both treatment-seeking and non-treatment-seeking populations. We first examine the relationship between control over AVH and health status as well as the psychosocial factors that may influence control and functioning. We then link control to various cognitive constructs that appear to be important for voice hearing. Finally, we reconcile the possibility of control with the field’s current understanding of the proposed cognitive, computational, and neural underpinnings of hallucinations and perception more broadly. Established relationships between control, health status, and functioning suggest that the development of control over AVH could increase functioning and reduce distress. A more detailed understanding of the discrete types of control, their development, and their neural underpinnings is essential for translating this knowledge into new therapeutic approaches.

## Introduction

Faced with the seemingly unimodal nature of the pre-existing term *vision*, Esquirol^1^ first introduced the term *hallucination* to the nascent field of psychiatry as follows: “A person is said to labor under a hallucination or to be a visionary who has a thorough conviction of the perception of a sensation, when no external object, suited to excite this sensation, has impressed the senses”. Others of the French school quickly adopted the definition^2^, which was carried forward by the major psychiatric textbook writers of the twentieth century, including Jaspers^3^ and Ey et al.^4^, who required that a hallucination: (1) have the appearance of a sensory event and (2) produce conviction in its reality^5^. Only in the late twentieth century did some in the field begin to define hallucinations as being necessarily outside of voluntary control. In their seminal book on the subject, Slade and Bentall^6^ added a third requirement, namely that hallucinations not be “susceptible to being voluntarily directed or controlled by those who experience [them].” This was in keeping with an understanding of hallucinations as being distinct from voluntary imagery^7^ or the pseudohallucinations of neurological illness, which, in addition to being amenable to initiation or interruption by will^3^, were described as often exhibiting a oneness of identity with the hearer, lacking the perceptual detail characterized by true hallucinations and necessarily occurring in the context of full insight into their unreality^8^.

Only recently have many researchers begun to recognize the possibility that phenomenologically rich, real-seeming auditory verbal hallucinations (AVH) can, in some cases, be voluntarily controlled. The advent of this work was made possible in part by the recognition that many of those who hear voices may never seek treatment^9–19^. Epidemiological studies suggest that 7–15% of the general population hears voices, at times regularly^20–22^, and only 20% of those who experience psychotic experiences (including AVH) go on to develop a psychotic disorder^22^.

Some have suggested that all voice-hearing experiences lie on a continuum^23^, while others argue that the experiences of treatment-seeking and non-treatment-seeking voice hearers are fundamentally different^24^, and still others suggest the possibility of multiple, potentially discontinuous continua^25^, perhaps defined by a separable factor coding for overall distress or dysfunction^26^. AVH in treatment-seeking and non-treatment-seeking individuals tend to be similar in terms of low-level acoustic qualities such as loudness, location, duration^27–29^, but show key differences in higher level, attributional characteristics such as interpretation of the voices’ origins, their perceived malevolence, and their ability to be engaged meaningfully^23,29–31^.

Non-treatment-seeking voice-hearing populations also consistently endorse a higher degree of control over their experiences than their treatment-seeking counterparts^23,27,29,30,32–34^. Perhaps most strikingly, some individuals in non-treatment-seeking groups report an ability to control the onset and offset of their voices^29,35,36^, which may make the experience of living with these voices significantly less disruptive and distressing^37^. Further, narratives surrounding the development of these abilities highlight the possibility that they may be intentionally nurtured and developed over time^29,38^.

Although control over AVH has been reported repeatedly in the literature, there has been little in-depth examination of its meaning, its development, or its cognitive, computational, and neural bases. Here we present an overview of the field’s current understanding of control over AVH in both treatment-seeking and non-treatment-seeking populations. We begin by discussing the concept of control over AVH and the varieties of control seen in the literature. We then provide an overview of the relationship between control and functioning in voice-hearing populations, followed by an examination of the psychosocial and cognitive factors that appear to influence control. We then attempt to reconcile the possibility of control with the field’s current understanding of the proposed cognitive, computational, and neural underpinnings of hallucinations and perception more broadly. Lastly, we consider therapeutic implications of the systematic study of control over AVH.

## Varieties of control

**Fig. 1:**
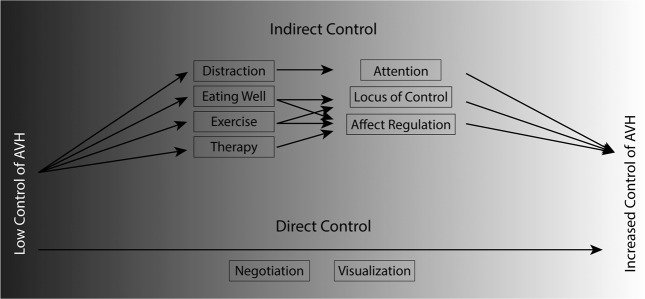
Spectrum of control over hallucinations. Phenomenological descriptions of degree of control over hallucinations in voice-hearing populations include a large spectrum of abilities. Some may be classed as “indirect control” abilities, which take advantage of the relationships that exist between domains that may be manipulated (like attention and overall sense of wellness and control over one’s life) and the potential for voices to impact the voice hearer negatively. Others may be described as “direct control” abilities, which use various techniques to directly influence voices’ onset and offset. These abilities have been described in several different populations and likely rely on different cognitive abilities and computational and neural architectures. All appear to be amenable to purposeful development. We argue that all of these abilities are likely captured by commonly used clinical rating scales. However, a fuller understanding of overall control’s component parts may be important for development of novel treatment strategies based on their cognitive or neural underpinnings.

Control of AVH may be defined as an ability to voluntarily influence one’s voice-hearing experience. It is clear from the literature that control over one’s hallucinations, like hallucination itself^23,25^, cannot be considered a unitary phenomenon. Rather, it appears to exist as a variety of abilities within a myriad of contexts. These abilities seem to fall into broad categories: direct control strategies and indirect control strategies (Fig. 1). Indirect control strategies include those that influence the frequency or impact of one’s voices by manipulating other related factors like attention. Direct control describes the ability to influence the onset and offset of these experiences by interacting with them more directly (e.g., negotiating boundaries with the voices or telling the voices to go away^35^). The ability of some voice hearers to exercise indirect control by using cognitive strategies to manipulate the impact of voice hearing has served as the basis for cognitive–behavioral approaches to psychosis for several decades^39,40^. However, the ability to directly control their onset and offset^29,35,36^ has not played a prominent role in traditional therapeutic approaches in the United States.

The literature surrounding control over hallucinations seldom specifies the type of control endorsed by participants, focusing instead on degree of perceived control endorsed by the participant on clinician-rated scales like the PSYRATS-AH^41^ (see Table 1). Potentially related forms of cognitive control have also been assayed, but none have focused on control over hallucinations themselves^42^. The authors of the PSYRATS note that reliability of the PSYRATS control item is unusually low “due to the complexity of control as a construct.” The authors go on to state that other instruments might better capture important determinants of control. We agree. We will argue that type and degree of endorsed control in voice-hearing populations should be defined carefully and specifically in the future, as these different types of control are likely subserved by different cognitive and neural mechanisms and may drive the development of vastly different treatment strategies.

**Table 1:** Descriptions of voluntary control in the literature.

Authors	Year	Study design/type	Participants	Type of control described
Powers, Kelley, Corlett	2017	Quantitative: questionnaire-based Qualitative: semi-structured interview	*N* = 16 participants with a psychotic disorder with AVH; *N* = 16 participants with a psychotic disorder without AVH; *N* = 17 non-clinical participants with AVH; *N* = 18 nonclinical participants without AVH	*Direct control*: clairaudient psychics able to control the onset and offset of voices
Roxburgh and Roe	2014	Qualitative: interviews	*N* = 10 spiritualist mediums	*Direct control:* mediums describe ability to “prevent or assist” communication
Taylor and Murray	2012	Qualitative: interview	*N* = 6 mediums	*Direct control:* mediums are able to choose when to engage with spirits
Jackson, Hayward, Cooke	2011	Qualitative: interview	*N* = 5 NHS service users with AVH; *N* = 7 non service users with AVH	*Direct control*: asserting boundaries through use of sprit guides, visualization, used to increase control
Knols and Corstens	2011	Qualitative: case study of treatment with Maastricht approach	*N* = 1 individual with AVH	*Direct control*: therapy aimed at helping patient understand meaning of voices helped him gain control over them
Chadwick and Birchwood	1994	Qualitative: interview	*N* = 25 participants with schizophrenia	*Direct control:* some participants able to control onset or offset of voices
Hutton, Morrison, Taylor	2012	Qualitative: case study	*N* = 1 individual with distressing and dominating AVH	*Direct/indirect control*: CBT associated with decrease in dominance of AVH, ultimate disappearance of AVH
Gottlieb et al.^137^	2013	Quantitative: questionnaire based (PSYRATS)	*N* = 17 individuals with schizophrenia spectrum disorder, AVH	*Indirect control:* greater perceived control over voices after completing CBTp
Falloon and Talbott	1981	Qualitative: interview	*N* = 40 schizophrenia outpatients	*Indirect control:* some voice hearers can use cognitive strategies to manage voices
Bentall et al.	1994	Patients assessed before and after receiving focusing therapy	*N* = 6 patients with schizophrenia	*Indirect control:* focusing therapy can be used to manage voices
Peters et al.	2012	Quantitative: questionnaire based	*N* = 46 participants at an outpatient psychosis clinic	*General control*: resistance to voices correlated with higher levels of perceived omnipotence of voices
	2003	Quantitative: questionnaire based.	*N* = 75 schizophrenia patients	*General control*: attempts to resist or block voices led to greater perceived intrusiveness
Chadwick et al.	2000	Quantitative: questionnaire based	*N* = 18 patients experiencing drug-resistant, distressing AVH	*General control:* CBT led to reduction in appraisals of voices as omnipotent
Honig et al.	1998	Quantitative: semi-structured interview	*N* = 18 schizophrenia patients with AVH; *N* = 15 Dissociative disorder patients with AVH; *N* = 15 non-clinical participants with AVH	*Perceived/rated control (NOS):* non-clinical voice hearers felt more in control of hallucinations than clinical voice hearers on self-report
Daalman et al.	2011	Quantitative: task based	*N* = 118 clinical participants with AVH; *N* = 111 non-clinical participants with AVH	*Perceived/rated control (NOS):* greater controllability of voices in non-psychotic individuals with AVH as rated on PSYRATS
Sorrell, Hayward, Meddings^138^	2010	Quantitative: questionnaire based	*N* = 32 clinical voice hearers *N* = 18 non-clinical voice hearers	*Perceived/rated control (NOS):* greater emotional distance from voices in those with clinical AVH than non-clinical AVH as rated on PSYRATS

Note that, while several qualitative studies have highlighted the existence direct control abilities, most quantitative studies have focused on either coping strategies or more general control abilities as rated by participants or by clinicians on general scales like the PSYRATS.

Interestingly, different types of control appear to be present across a wide variety of voice hearers. Ability to control the onset and/or offset of voice hearing through direct interaction with voices (*direct control*) appears to be endorsed frequently in those who interpret their voice hearing spiritually. Classic ethnographic studies by Murphy (1976) describe shamanistic practices among Inuits, comparing these with individuals recognized to be mentally ill in the same population. In making the distinction between these two groups, Murphy contends that any such distinction depends on “the degree to which they are controlled and utilized for a specific social function. The inability to control these experiences is what is meant by a mind out of order; when a mind is out of order it will not only fail to control sensory perception but will also fail to control behavior”^24^. More recently, Powers et al.^29^ compared treatment-seeking individuals with AVH to self-identified clairaudient mediums and found that the latter population was more likely to report being able to summon or stop their AVH at will. Similarly, a recent qualitative analysis of the experiences of ten British Spiritualists found that these individuals described a similar ability to “shut off” their voices in order to prevent them from interfering with their lives^35^. Another study of spiritualist mediums reported similar findings^36^. As one participant in the latter study described it: “When you’re working, you’re working, and when you’re not, you’re not. Say to the spirit that, you know, I’ll be there Thursday at 7:30”^36^. In clinical populations, development of such direct control abilities has been reported in voice hearers under guidance of the Hearing Voices Movement (HVM)^43,44^ for decades. Development of these abilities, while described briefly in small studies as requiring engagement and practice^29,35^ and implicitly acknowledged as being effortful in HVM guidelines^43–45^, have yet to be outlined in detail. The process by which these skills are fostered and developed carry obvious implications for the development of treatment based on these findings. In the next section, we begin to outline potential factors that may relate to the development of these abilities.

## Control, health status, and functioning

Regardless of type, degree of perceived control over the voice-hearing experience appears to be critical for the level of distress or dysfunction experienced by the voice hearer. There have been several qualitative and quantitative studies directly comparing the experience of treatment-seeking and non-treatment-seeking populations with AVH in which control emerges as a distinguishing feature^12,23,30,41^. Birchwood et al.^46^ first identified that distress among patients with psychosis is potentially related to a perceived lack of control over their illness, including hallucinations. After developing a comprehensive interview aimed at understanding the experiences of voice hearers across the spectrum of illness, Romme and Escher^47^ reported that the majority of non-treatment-seeking voice hearers (and some treatment-seekers) felt in control of their voices and experienced positive feelings about their content.

Larøi et al.^12^ also identified perceived control as being potentially important for functioning in a study of 236 university students who also completed standard measures of hallucination frequency and intensity, finding that affective response to hallucination and ability to control one’s experiences were significantly associated. Daalman et al.^27^ compared patients and non-patients with AVH using the Psychotic Symptom Rating Scales—Auditory Hallucinations Subscale (PSYRATS-AH)^41^ and found greater control over AVH in non-patients than in patients. Similarly, Honig et al.^34^ interviewed patients and non-patients with AVH and found that non-patients reported feeling more in control of their hallucinations than did their clinical counterparts. These findings are extremely common in descriptions of non-treatment-seeking voice hearers^12,23,27,29,30,32,34^: a recent systematic review found that, of the 12 studies identified at that time to have compared perceived control in these two groups, 10 found non-treatment-seekers to have higher endorsed control, while 2 showed no difference^23^. These differences in control are not always seen at the initial onset of AVH, with several studies of non-treatment-seeking voice hearers finding that control is most frequently developed intentionally over time^35,38^.

## Psychosocial influences

### Locus of control

The degree to which one feels one has control over one’s life in general may play a role in the amount of control one experiences in regard to AVH. One measure of perceived control is the locus of control scale, which measures the degree to which one perceives one’s life to be controlled by outside forces (external) vs. by one’s own choices and actions (internal)^48^, with an individual’s locus of control lying somewhere on a spectrum between these two points. Studies have found that individuals with a psychotic disorder have a more external locus of control than the general population^49^. A longitudinal study found that a more external locus of control in adolescence strongly predicted a diagnosis of schizophrenia as an adult^50^. Relatedly, a qualitative study of voice hearers both with and without a psychiatric diagnosis found that “developing a stronger sense of self and independence,” was a crucial part of learning to live with voices^51^; a similar stance is explicitly endorsed by the HVM^43–45^.

A variety of factors may moderate the relationship between health status and locus of control. Post-traumatic stress disorder (PTSD), for instance, is also associated with a more external locus of control^52^. Both treatment-seeking and non-treatment-seeking AVH populations show higher levels of trauma than the general population^21,53^, but those in the former group are more likely to have symptoms sufficient for diagnosis with PTSD^32^. Even among non-treatment-seeking voice hearers, a more external locus of control is weakly predictive of severity of hallucinations and delusion-like beliefs^54^. However, further research into the relationship between locus of control and control over AVH is needed to determine the causal direction of this relationship.

### Engagement vs. resistance

It has been suggested that engagement, a willingness to interact with voices rather than attempting to ignore them or block them out, may be predictive of control over AVH. This involves various forms of interaction such as discussion, negotiation, and boundary setting, and often involves attributing some form of agency or personhood to voices. Engagement may be contrasted with resistance, or a refusal to acknowledge or interact with voices^55,56^. Several studies suggest that engagement with voices increases perceived control over voices and resistance decreases it. Peters et al.^56^ found that resistance to voices was correlated with higher levels of perceived omnipotence of those voices. Omnipotence was also related to a greater degree of distress related to voices. Others have reported that participants endorse higher degrees of control after undergoing spiritually oriented training in which engagement with voices is encouraged^35,36^. Interestingly, participants also reported that ceasing to engage with voices made their experiences worse, further suggesting that engagement may be important for the maintenance of control.

One study demonstrated that those without a diagnosed psychiatric disorder were more likely to engage with voices than those with a diagnosed psychiatric disorder^57^, highlighting a potential role for the content of voices and the distress they evoke in influencing likelihood of engagement. Negotiation, boundary setting, and compromise are another form of engagement. Luhrmann (2012) describes an individual whose voices instruct him to become a Buddhist. At the urging of his HVM group, he tells the voices that, each day, he will spend 1 hour reading about Buddhism and say one Buddhist prayer if they will leave him alone. The voices do begin to leave him alone, he is able to reduce his medication, and, ultimately, he returns to normal functioning^38^.

Others have examined the notion that the ways in which individuals interact with their voices may be similar to the dynamics of relationships with the people in their lives^58^. Related to the broader concept of locus of control, Birchwood et al.^59^ found that those who related to their voices from a position of powerlessness and subordination were more distressed by them than those who did not. Hayward^58^ found that those who felt distressed by their voices were likely to feel powerless in relation to them and, furthermore, reacted to this by seeking distance from their voices. Qualitative research has also found that, for some voice hearers, ignoring voices for long periods of time can make them louder and perceived more externally^60^. Overall, then, it appears that suppression of voices is correlated with negative outcomes and engagement with positive outcomes. This relationship could be related to the tendency for thoughts one has attempted to express to become more cognitively available and further strain already limited cognitive inhibitory resources, as proposed by Badcock^61^.

### Beliefs about voices

The impact of voices on one’s sense of control, as well as overall functioning, may be influenced by voice hearers’ beliefs about these voices and the amount of power that they may exert^62^. A tendency to appraise AVH as powerful has been shown to correlate with likelihood of following command hallucinations^63^, and treatment-seeking voice hearers report more negative beliefs about the danger and uncontrollability of voices^57^. The explanatory framework one applies to understand one’s voices also appears to have an impact on one’s perceived level of direct control over AVH. Qualitative work suggests that learning to view voice-hearing experiences within a spiritualist framework may lead to relief from distress associated with these voices^35^.

### Metacognition

The ways in which individuals respond to their thoughts and AVH may interact with control over them. Brett et al.^64^ found that metacognitive beliefs regarding a need for control were associated with more negative responses to anomalous experiences. A follow-up study used the Meta-Cognitions Questionnaire (MCQ)^65^ to demonstrate significantly higher levels of metacognition concerning a need for control in clinical versus non-clinical voice hearers^66^. It may be that the need for control over one’s own thoughts is related to resistance to AVH, which has also been shown to be negatively correlated with control over AVH and positive health outcomes. This further suggests that comorbid factors such as depression may play a significant role in differentiating treatment-seeking and non-treatment-seeking populations with AVH.

### Intentional inhibitory control

Several studies have found that AVH is associated with decreased intentional inhibition^42,67^. Those with AVH do not, however, differ from non-hallucinating controls on measures of automatic^42^ or interference control^68^. Proneness to auditory hallucinations is correlated with deficits in intentional inhibition of intrusive thoughts, in both those with schizophrenia^67^ and healthy individuals with a high predisposition to hallucination^42,69^. The degree of intentional inhibitory dysfunction has also been found to be correlated with the frequency of auditory hallucinations in patients with schizophrenia^67^. Future work should explicitly examine the relationship between ability to control AVH both directly and indirectly and intentional inhibitory control.

### Other factors

Spiritually oriented voice hearers may employ other techniques to promote development of control over AVH. Mediums may use a “spirit guide,” for instance, to help them manage the voices^29,36^. Calling in spiritual help was also found to be a useful technique for controlling voices in a study of voice hearers both with and without a psychiatric diagnosis^51^. This study, which utilized grounded theory to qualitatively analyze the participants’ experiences of voice hearing, also found that visualization was utilized to control voices: some participants reported visualizing an energy field around them which the voices could not cross. In Taylor and Murray’s^36^ qualitative study of mediums, participants describe several techniques which helped them gain control over voices, including meditation. Another participant describes a visualization exercise, consisting of imagining a light around her which the voices cannot penetrate as helping her to control the voices. A more focused analysis of these techniques and their potential to facilitate direct control over AVH is needed.

The need for control over one’s thoughts may be influenced by cultural assumptions about the mind. For example, in a culture that assumes that the mind is not permeable, hallucinations would represent a more radical rupture of self and their uncontrollable nature would be more threatening. Indeed, qualitative research suggests that participants with psychosis in India and Ghana, where boundaries of the self may be more permeable^70^, are less disturbed by their inability to control voices^71^.

## Potential cognitive, computational, and neural mechanisms

If voice hearers can exert some degree of control over their experiences with AVH, how might this work in the context of what we understand to be the potential cognitive, computational, and neural mechanisms underlying AVH? We briefly consider the implications of control over hallucinations in the context of three dominant neuroscientific theories of hallucination: corollary discharge, misattribution of inner speech, and Bayesian models.

Conceptualization of hallucinations as uninhibited, externalized thoughts fits well with some methods of control outlined above. One popular account of AVH views these phenomena as a disturbance of corollary discharge, a process by which information on planned, self-generated actions is used to predict the sensory consequences of those actions. Intact corollary discharge results in an attenuation of the somatosensory response arising as a consequence of one’s own actions, for example: reaching out to touch a ball with one’s hand generates a much less robust somatosensory response than if the ball were to hit one’s hand. Thus, extant knowledge about the location and movements of one’s own body allows for prediction and partial cancellation of the sensory consequences of one’s actions, and the absence of such a cancellation implies that these sensations are the result of being *acted upon* rather than *acting*. These processes have been heavily implicated in causal inference^72^. It has been proposed that a failure to predict the sensory consequences of one’s actions could lead to misattribution of inner speech to an external source^73,74^. Indeed, people with schizophrenia fail to predict the sensory consequences of their actions in somatosensory^75^, visual^76–78^, and auditory^74,79–82^ sensory modalities. Some have hypothesized that failure to predict the sensory or neural consequences of internal speech may produce hallucinations, taking as supporting evidence the smaller mismatch negativity amplitudes and a host of other failures of prediction typically seen in schizophrenia^73,74^ (for review, see ref. ^83^).

Indirect control strategies shown to reduce the impact of voices via attentional allocation may serve to shift focus away from internally generated thought patterns considered to be involved in the generation of AVH. This may be related to general cognitive control abilities. Research has found that treatment-seeking voice hearers experience more intrusive thoughts than those with non-clinical AVH^84^ and that intentional inhibition—the ability to block unwanted thoughts from arising—is related to propensity to hallucinate in the general population^69^ and severity of hallucinations in clinical groups^85^. Others have similarly shown that patients with schizophrenia demonstrated less inhibitory control over irrelevant memories than healthy controls^67,86^, which may in turn be dependent on hippocampal GABAergic function^87^. These findings may form the basis for an understanding of how inner-speech-based frameworks may account for direct control.

Similarly, both direct and indirect control may be accounted for by Bayesian accounts of voice hearing, which attempt to place hallucinations in a common framework with the mechanisms of everyday perception. These frameworks conceive of perception as an iterative process of *unconscious inference*, in which we automatically infer what is around us by combining our sensory input with our prior beliefs about the world^88,89^. This blending of prior beliefs and sensory input is observed readily in many daily situations, from the use of lip-reading cues^90^ and sentence context in understanding speech in auditory noise^91^ to the use of shading for depiction of depth in visual art^92^. Bayesian statistics have been used to construct models describing how this combination of sensory input and prior beliefs takes place^93^. These models have succeeded in predicting performance on a wide range of perceptual tasks^94–96^ as well as the activity of single units and ensembles of neurons in sensory cortices^97^. Within this predictive coding framework, hallucinations may result from overly-weighted perceptual beliefs. In the setting of increased cortical noise (as in psychosis^98^), reliance on prior expectations within an auditory system tuned to detect the human voice^99^ may be adaptive.

Converging evidence from several different paradigms has highlighted this over-weighting as being critical in distinguishing participants with and without hallucinations^100–103^. Powers and colleagues used a Pavlovian conditioning procedure to produce detection of an auditory target despite its absence, contingent upon the presence of a visual stimulus (i.e., a conditioned auditory hallucination). Performance on the task demonstrated a fivefold increase in reporting conditioned hallucinations in voice hearers compared to non-voice hearers, and modeling of behavior on the task revealed that this increase was due to hyper-precise priors in voice hearers^101^. Similarly, Alderson-Day and colleagues showed that non-clinical voice hearers demonstrate an enhanced ability to recognize sine-wave speech compared to non-voice hearers, interpreted as an increased ability to use priors in ambiguous perceptual contexts. A related effect was demonstrated by Teufel et al.^102^ in the visual realm. Lastly, Cassidy et al.^104^ demonstrated that voice hearers were likely to make more use of prior expectations in judging the duration of an auditory stimulus under different levels of uncertainty.

As with the inner-speech hypothesis, attentional-reallocation strategies used by those who exhibit indirect control may be explained within the predictive coding framework. In this case, a shift in attention to environmental stimuli may result in an increased precision of sensory evidence^105^, leading to a decrease in the relative weighting of priors and a subsequent decreased propensity to hallucinate.

Direct control may also be explained within the context of the predictive coding framework. Within the perceptual hierarchy, precision of priors at any particular level depends on higher-level beliefs about the reliability of that information^106^. Thus, higher-level expectations about the relationship between contextual signals and the reliability of perceptual priors may allow for dynamic modulation of the precision of those perceptual priors^107^. Engagement with voices may allow for learning and manipulation of these contextual relationships. Indeed, phenomenological descriptions of the development of direct control abilities focus on the development of a relationship with one’s voices in the service of establishing mutually defined expectations around when and how voices may engage with the voice hearer^29,35,43–45,108^. Thus, development of direct control over one’s voices may result from the influence of learned social and contextual expectations arising from direct engagement with them. If this is true, individuals with better social cognitive abilities may exhibit enhanced direct control compared to those with poor social cognitive skills. Social expectations themselves are learned from one’s past social experiences; thus, the use of social expectations to inform precision of perceptual priors may explain some of the known relationships between trauma exposure, locus of control, agency, and ability to control one’s hallucinations^109^. Indeed, past trauma has been shown to affect processes related to detection of basic auditory stimulus features^110^. These processes also change with affective state^111^. Relatedly, unusual experiences have also recently been linked to absorption and social expectations^112^, providing some evidence that such high-level social expectations can be linked not only to perception in the laboratory, but unusual perceptual experiences as they exist in clinical settings.

An alternative to direct manipulation of prior precision during direct control could be calibration of volatility beliefs regarding the same contextual relationships. A recognition that previously learned associations may be changing over time may lead to an adaptive alteration in the precision of priors relative to sensory information, as is seen in some non-treatment-seeking voices hearers and likely involves structures such as the cerebellum, known to be involved in model-based processing^101^. A more direct pathway toward direct control may also arise from engagement with voices, via more low-level perceptual learning mechanisms. The relationships between direct control, priors, and incoming sensory evidence may perhaps be examined using stimuli for which multiple valid perceptual interpretations may apply. This is the case in bistable or multistable perception, which is susceptible to direct, top-down control^113^ and has known neural correlates^114^. Crucially, these may be similar to those of high-level Bayesian perceptual inference^101,115–118^.

Extension of Bayesian perceptual models toward the inclusion of voluntary action may also provide a new window of opportunity for understanding voluntary control over hallucinations. While traditionally thought of as distinct, informationally encapsulated processes^119^, perception and action are explicitly related in recent formalized descriptions of perceptual processing. These active inference-based models extend the same principles of free-energy minimization underlying the Bayesian models above toward action^120^. Although initially proposed to account for the influence of action on perception^121^, recent formulations have proffered valuable extensions toward explanation of diverse phenomena of direct relevance to neuropsychiatry, including action selection^122^, alterations of consciousness^123,124^, and psychosis^125^. These models have been extended to provide a compelling formal account of agency and cognitive control^126–128^. Application of mechanisms accounting for agency toward those components involved in basic perceptual inference would likely produce specific hypotheses regarding the development and mechanisms underlying control over hallucinations.

In general, it may be that different sub-populations of voice hearers exhibit different types of computational deficits. If this is the case, these different subgroups may also vary in their control abilities in a way that reflects their underlying deficits. Lastly, it should be noted that recent advances in neurofeedback training for treatment of auditory hallucinations appear to offer a theory-agnostic demonstration of direct control development^129,130^. In fact, the strategies participants use during neurofeedback sessions appear to be mostly implicit and perceptually-based (see ref. ^130^, Supplement), implying that a perceptual component independent of higher-order cognitive strategy may be isolated from psychosocial factors that influence direct control development. Thus, future work may choose to refine these methods by specifically altering perceptual circuits shown to be involved in the execution of direct control over AVH.

## Therapeutic applications

Many extant approaches to treatment of hallucinations may enhance indirect control. They generally involve the development of active cognitive coping strategies and tools consistent with cognitive–behavioral therapy (CBT)-based treatment. Attention training, a technique aimed at increasing attentional control^131^, has been shown to increase perceived control over AVH in one case study^132^. Chadwick and Birchwood (1994)^55^ reported a very low rate of perceived control over voices among a clinical voice-hearing sample, but also described a higher degree of endorsed control after therapy, which correlated with other measures of functional improvement after intervention. They later demonstrated that therapy may result in decreases in beliefs about voice omnipotence, but fails to produce improvement in affect^133^. CBT-based approaches may also result in harm reduction in those with command hallucinations, likely driven by decreased beliefs about the omnipotence of voices^134,135^.

Some treatment strategies also work on direct control through interaction with voices. The Maastricht Approach^136^, for instance, which involves examining the meaning and origins of the voices and learning to fit them into a coherent life narrative. Some cases demonstrate the ability of this reframing to produce new, more positive relationships with one’s voices^108^.

Although intentional manipulation of explanatory frameworks around voice-hearing experiences and with negotiation with voices may form the basis for new psychotherapies aimed at enhancing agency and control in voice hearers, an understanding of the computational and neural underpinnings of direct control may lead to a harnessing of these processes for biological interventions. For example, pharmacological, neurofeedback, and Repetitive transcranial magnetic stimulation (rTMS)-based interventions may result from an understanding of model parameters and brain regions involved in exerting direct control over voices. Lastly, unlike symptom-focused approaches to treatment aimed at decreasing or eliminating voice hearing, control-based approaches would reframe treatment toward recovery and development of agency over symptoms in voice hearers.

Treatment approaches based upon enhancing control over one’s voices need not conflict with conventional psychiatric treatment. In fact, these strategies may be some of the most viable options for treatment in individuals who suffer from hallucinations but for whom the risks of antipsychotic medications may outweigh the benefits. These populations, including individuals with treatment-resistant AVH and young people at clinical high risk for psychosis, may be particularly suitable to treatments based upon the findings outlined here.

## Conclusions

The ability to exert some degree of control over aspects of one’s auditory hallucinations may be important for long-term outcomes and overall functioning. This observation, while repeated time and again in the literature, will be difficult to translate to new treatments without a more detailed understanding of the varieties of control that may be exerted. Those interested in exploring these abilities may benefit from an initial participant-driven, qualitative approach to the phenomenology of control. This may focus on the spectrum of control abilities as well as on social, demographic, clinical, and perceptual factors contributing to control.

One of the fundamental questions raised by the co-existence of treatment-seeking and non-treatment-seeking voice hearers is that of whether an individual member of one group could have been part of the other had circumstances been different. Or, could an individual currently in one group transition to the other? There is a small amount of evidence to suggest that the development of control abilities may increase functioning: development of control may actually facilitate a change in health status^38^. However, there is clearly a need to study the development of control over AVH in much greater volume and detail. This should be conducted along with in-depth characterization of voice-hearing experiences to determine if the difference between clinical and non-clinical voice-hearing populations is one of kind or of circumstance.

There is also clearly a need for a tool to quantify voice hearers’ abilities across the various domains of control. Ideally, such a tool would allow for capture of these abilities at the time of appraisal but also at time of initial voice hearing. Doing so would allow for a staging of control abilities and targeted intervention. Breaking perceived control down into more well-defined abilities may allow for more specific targeting of the cognitive, computational, and neural processes underlying these abilities as well as case-specific staging and intervention based upon the deficits exhibited by each person seeking care.
